# ADVANCE CARE PLANNING FOR SPANISH-LANGUAGE SPEAKERS: PATIENT, FAMILY, AND INTERPRETER PERSPECTIVES

**DOI:** 10.1093/geroni/igac059.1858

**Published:** 2022-12-20

**Authors:** Geraldine Puerto, Germán Chiriboga, Susan DeSanto-Madeya, Vennesa Duodu, Dulce Cruz-Oliver, Jennifer Tjia

**Affiliations:** University of Massachusetts Chan Medical School, Worcester, Massachusetts, United States; University of Massachusetts Chan Medical School, Worcester, Massachusetts, United States; University of Rhode Island, Providence, Rhode Island, United States; University of Massachusetts Chan Medical School, Worcester, Massachusetts, United States; Johns Hopkins Medical School, Baltimore, Maryland, United States; University of Massachusetts Chan Medical School, Worcester, Massachusetts, United States

## Abstract

Language access is a challenge to advance care planning (ACP). Spanish-language speakers are the largest non-English speaking population in the US. While ACP tools have been translated into Spanish, it is unclear how heterogeneity in country of origin may affect the generalizability of translations across diverse US Spanish-speaking populations. The study objective is to describe challenges and facilitators to ACP for diverse populations of Spanish-language speakers. We conducted 3 focus groups with a total of 29 participants from members of Spanish-language speaking communities whose countries of origin were predominantly from the Caribbean, Central, and South America. Eligibility included being 18 years or older, being a native Spanish-speaker, and having direct experience with ACP as a patient, caregiver, or medical interpreter. We conducted thematic analysis with axial coding. Themes include: 1. Linguistic Challenges with Current ACP Translations; 2. Effect of Country of Origin and Culture on ACP Understanding; 3. Impact of Local Healthcare System Cultural on ACP; and 4. Need for ACP to be Normalized into the Local Community. ACP is both a cultural practice and a clinical practice. Recommendations for improving ACP completion for non-English speakers extend far beyond translation, since simply translating ACP tools without a cultural context is neither equitable nor inclusive. A key step is normalizing ACP into the local community. Understanding the intersection of local healthcare systems of ACP practice with the patient’s and family’s cultures of origin will facilitate introducing ACP in a culturally sensitive manner.

